# Evaluation of renal circulation in heart failure using superb microvascular imaging, a microvascular flow imaging system

**DOI:** 10.1007/s10396-023-01397-6

**Published:** 2024-01-18

**Authors:** Shohei Kikuchi, Kiyomi Kayama, Yu Kawada, Shuichi Kitada, Yoshihiro Seo

**Affiliations:** Department of Cardiology, Nagoya City University Graduate School of Medical Sciences, 1 Kawasumi, Mizuho-cho, Mizuho-ku, Nagoya, 467-8601 Japan

**Keywords:** Heart failure, Cardiorenal syndrome, Renal circulation, Renal ultrasound, Microvascular imaging

## Abstract

**Purpose:**

Renal circulation evaluation is essential in understanding the cardiorenal relationship in heart failure (HF), and there is a growing interest in imaging techniques that visualize renal circulation. This study aimed to assess the effectiveness of superb microvascular imaging (SMI) in evaluating renal circulation in HF patients.

**Method:**

The study included 71 HF patients undergoing cardiac catheterization. Prior to catheterization, renal ultrasound examinations were performed. A control group of 18 subjects without HF was also included. SMI was used to measure the vascular index (VI), which was calculated as the percentage of blood flow signal area in the region of interest. The intrarenal perfusion index (IRPI) was determined as a fluctuation index of VI, reflecting variations in the number of blood cells moving through renal tissue during the cardiac cycle.

**Results:**

Using the upper 95% confidence interval of IRPI (0.6) from the control group, HF patients were classified into two groups. Patients with IRPI > 0.6 showed a more congestive profile. Right atrial pressure and biphasic or monophasic Doppler intrarenal flow pattern were independent determinants of IRPI > 0.6. In addition, IRPI remained a significant predictor of estimated glomerular filtration rate (eGFR).

**Conclusion:**

The parameter IRPI as variations in SMI signal during the cardiac cycle may be a useful evaluation method for renal perfusion impairment in HF.

## Introduction

The close relationship between the heart and kidneys is known to be involved in the development and progression of cardiac diseases, known as cardiorenal syndrome (CRS) [1]. From a hemodynamic perspective, in addition to reduced cardiac output due to left ventricular (LV) pump failure, renal congestion due to right heart failure (HF) and fluid retention also contributes to worsening renal function [2, 3]. Although the presence of renal congestion has been indirectly estimated using elevated central venous pressure (CVP), we proposed the use of Doppler intrarenal venous flow (IRVF) at the renal interlobar veins to assess renal congestion in HF patients [4]. In normal subjects, the Doppler waveform of IRVF was recorded continuously throughout one cardiac cycle, but in some patients with HF, discontinuous IRVF patterns were observed, such as biphasic and monophasic Doppler waveforms, and we found that HF patients with discontinuous IRVF patterns had a poorer prognosis. Subsequently, various studies have confirmed the clinical usefulness of IRVF in HF [5–8], and discontinuous IRVF patterns have been recommended as a marker of renal congestion [9].

HF increases vasopressin-mediated water reabsorption, but venous stasis prevents this absorption, resulting in edema of the renal medulla. The medulla contains straight blood vessels surrounding the renal tubules, and the increased renal interstitial pressure caused by edema results in the compression of the straight blood vessel bundles, resulting in impaired medullary blood flow, leading to ischemia that worsens renal function. In addition, tubular pressure increases due to tubular compression, and Bowman’s capsule pressure rises. The filtration pressure between the glomerular capillaries and Bowman’s capsule decreases, resulting in a decrease in glomerular filtration rate and worsening renal function. Thus, renal congestion causes renal dysfunction through a complex mechanism, but it mainly occurs in the renal medulla. We have reported that IRVF is indirectly associated with microcirculation in the renal medulla, the primary site of renal congestion, in a rate model [10], but a more direct method of assessing the hemodynamics of the renal medulla may be more accurate in assessing the pathogenesis of renal congestion. Previously, we reported that contrast-enhanced ultrasound is a promising diagnostic tool to assess renal microcirculation, but its clinical specifications are limited by time consumption and the use of ultrasound contrast agents [11]. Besides, the evaluation of venous blood flow alone does not consider the hemodynamics of the arterial system, another cause of impaired renal perfusion [7]. We then looked into the application of microvascular flow imaging (MVFI) using the superb microvascular imaging (SMI) technique to overcome these challenges. SMI employs advanced clutter suppression to reflect microflow information, enabling the visualization of minute vessels with slow velocities without the need for contrast agents [12–14]. In this study, we aimed to evaluate the utility of SMI in assessing renal circulation in HF patients.

## Materials and methods

### Study design

Patients hospitalized for treatment of HF or for cardiac catheterization study were prospectively enrolled. Renal ultrasound and echocardiographic examinations were performed just before cardiac catheterization studies. In addition, 18 subjects without HF were enrolled to obtain SMI data in a control group.

### Renal ultrasound studies

1. SMI

Renal ultrasound studies were performed with an Aplio i800 system (Canon Medical Systems, Tochigi, Japan). Using a variable-frequency convex transducer (1.5–6.0 MHz), the right kidney was studied. Patients were placed in the supine position and were asked to stop breathing at the end of expiration or breathe quietly if impossible during image acquisition. As to the settings for SMI, the color velocity scale was adjusted to 2.8 cm/s and the frame rate was greater than 55 Hz. Gain settings were optimized for each imaging.

2) SMI study

**Fig. 1:**
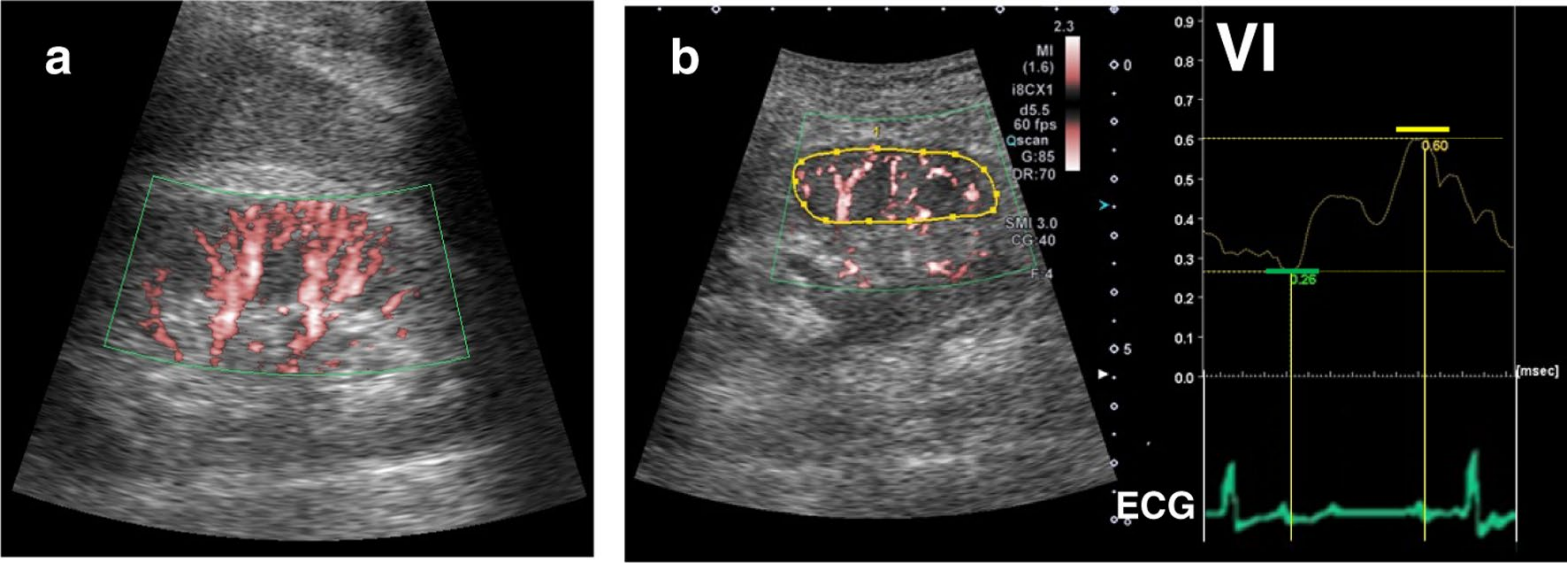
Quantitative evaluation of intrarenal blood flow using superb microvascular imaging. **a**: Intrarenal blood flow visualized on superb microvascular imaging (SMI). **b**: In the left panel, the area surrounded by the yellow curve is the region of interest. The right panel shows the time–vascular index (VI) curve. Based on the ECG, the maximum VI (yellow bar) and the minimum VI (green one) are determined in a cardiac cycle. The intrarenal perfusion index (IRPI) is calculated as the maximum VI minus the minimum VI divided by the maximum VI

SMI allows quantitative parameter analysis using the proportional parameter called vascular index (VI) to reflect the blood flow in the tissue [13]. VI was identified as the area percentage of blood flow in the focal lesion; VI = area of blood flow signal / area of region of interest (ROI). The ROI was set up to surround the renal cortex and medulla. Results from intrarenal Doppler (IRD) studies indicate that VI also changes over the cardiac cycle, and we hypothesized that the degree of change in VI may be greater in HF patients with renal congestion. Therefore, the intrarenal perfusion index (IRPI), as an index of cyclic variation in VI, was calculated as the maximum VI (Max.VI) minus the minimum VI (Min.VI) within one cardiac cycle in the ROI divided by the Max.VI (Fig. 1).

3) IRD study

IRD was recorded as previously reported [4]. Briefly, the velocity range of color Doppler was set to around 16 cm/s. Color Doppler images were used to determine interlobar vessels, and the sample volume was set based on the color Doppler signals derived from the interlobar arteries. Pulsed Doppler waveforms of the resistance index (RI) at a lobar artery was calculated as the maximum flow velocity minus diastolic flow velocity, divided by the maximum flow velocity. Doppler waveforms of IRVF were classified into three flow patterns: continuous, biphasic discontinuous, and monophasic discontinuous [4]. All measurements were averaged over three cardiac cycles during sinus rhythm. In patients with atrial fibrillation, an index beat, which was the beat following two preceding cardiac cycles of almost equal duration, was used for each measurement.

### Echocardiography

Comprehensive echocardiographic examinations were performed using the same ultrasound system. Experienced sonographers performed all echocardiographic measurements according to current guidelines [15]. LV end-diastolic volume (LVEDV), LV end-systolic volume (LVESV), stroke volume (SV), and LV ejection fraction (LVEF) were evaluated using the biplane disc method. Maximum left atrial (LA) volume was measured using the biplane Simpson’s method and indexed to body surface area (LAVI). Peak early (E) and late diastolic (A) velocities of the LV inflow and the average of peak early diastolic velocity on the septal and the lateral corner of mitral annulus (e′) were measured in the apical four-chamber view. The E/e′ ratio was obtained by dividing E by e′. Tricuspid regurgitation pressure gradient (TRPG) was derived from peak TR jet velocity. With the patient in the supine position, the diameters of the inferior vena cava (IVC) were measured in the subcostal view at 1.0 to 2.0 cm from the junction with the right atrium. The maximum diameter of the IVC and the percentage decrease in the diameter during inspiration were measured. Based on the guidelines, CVP was estimated using three grades consisting of 3, 8, and 15 mmHg.

### Cardiac catheterization

Cardiac catheterization studies were performed after the ultrasound studies. Right heart catheterization was performed with a 7Fr balloon-tipped pulmonary artery catheter (Swan-Ganz; Baxter Healthcare, Irvine, CA, USA). All pressure data were measured at end-expiration, with the reported values representing the average of 5–10 cardiac cycles. The cardiac index was measured using the thermodilution method. If a patient had pulmonary artery hypertension or significant tricuspid regurgitation, the cardiac index was measured using the Fick method. Aortic pressure (AP) was measured with a pigtail catheter in the ascending aorta.

### Laboratory data and renal function

Peripheral blood samples were taken just before echocardiographic studies. Estimated glomerular filtration rate (eGFR) was calculated according to the isotope dilution mass spectrometry Modification of Diet in Renal Disease equation modified for the Japanese population, i.e., eGFR (mL/min/1.73 m2) = 194 * (serum creatinine)^−1.094^ * age^−0.287^ (*0.739 if female). Plasma concentration of brain natriuretic peptide (BNP) was measured via chemiluminescence enzyme immunoassay (CLEIA) (Lumipulse^®^ Presto BNP; Fujirebio Inc., Tokyo, Japan) on the fully automated Lumipulse^®^ L2400 analyzer (Fujirebio Inc., Tokyo, Japan).

### Data variability

Two observers independently assessed Max.VI, Min.VI, and IRPI in 10 patients. To test intra-observer variability, a single observer analyzed the data twice on occasions separated by a 1-month interval. To test inter-observer variability, a second observer analyzed the data without knowledge of the first observer’s measurements. Reproducibility was assessed as the mean percent error (absolute difference divided by the mean of the two observations).

### Statistical analysis

Results are expressed as number (%) or as mean ± standard deviation (SD). Comparisons between two groups were performed using Student’s *t *test for continuous variables and the χ2 test for categorical variables. Correlations between variables were assessed using simple linear regression analysis. One-way analysis of variance (ANOVA) with the post hoc Tukey–Kramer test was used to compare variables between three groups. Independent determinants of IRPI greater than 0.6 were assessed by means of multivariable logistic regression analyses adjusted using univariate factors with a value of *p* < 0.05. Independent determinants of eGFR were assessed by means of multivariable regression analyses adjusted using univariate factors with a value of *p* < 0.05. All calculations were performed with SPSS ver. 28 (SPSS Inc., Chicago, IL, USA).

## Results

### Comparisons between control and HF groups

**Fig. 2:**
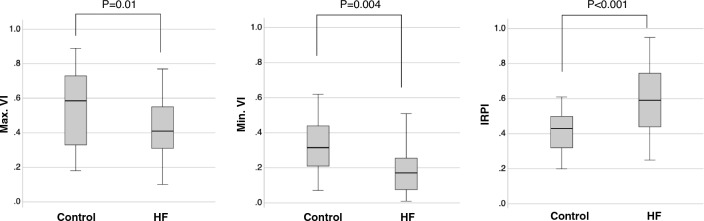
Comparisons of SMI data between heart failure patients and control subjects, *HF* heart failure, *Max.VI* maximum VI, *Min.VI* minimum VI, *IRPI* intrarenal perfusion index

The clinical characteristics of HF patients and control subjects are summarized in Table 1. Some control subjects had comorbidities including hypertension and diabetes, but significant abnormalities in laboratory data, cardiac function, and hemodynamics were not observed. In the HF group, 13 patients (18%) showed one or more systemic congestion signs including jugular vein distention, hepatojugular reflux, edema of the lower extremities, and pleural effusion at examinations. Comparisons of SMI data between the HF and control groups are shown in Fig. 2. Both Max.VI and Min.VI values in the HF group were lower than those in the control group. There was a strong correlation between Max.VI and Min.VI (correlation coefficient (*R*) = 0.790, *p* < 0.001). Nevertheless, IRPI separated the HF and control groups more significantly. This was due to a significant decrease in Min.VI compared to Max.VI, resulting in a larger difference between them in the HF group, the numerator of IRPI, compared to the control group (0.33 ± 0.10 vs. 0.23 ± 0.09, *p* = 0.01), and a decrease in Max.VI, the denominator of IRPI, resulting in a significant increase in IRPI.

**Table 1:** Comparisons of clinical characteristics between control and HF groups

Characteristic	Control	HF	*P* value
	(*n* = 18)	(*n* = 71)	
Age, yrs	62 ± 14	71 ± 11	0.006
Male	8 (44)	46 (65)	0.18
Body mass index	23 ± 4.5	22 ± 4.5	0.32
Systolic BP, mmHg	125 ± 19	120 ± 19	0.36
Diastolic BP, mmHg	73 ± 11	71 ± 12	0.55
Mean BP, mmHg	90 ± 12	88 ± 13	0.52
Heart rate, beats/min	68 ± 10	74 ± 14	0.09
Atrial fibrillation at the examination	0	19 (26)	–
Hypertension	6 (33)	41 (58)	0.07
Diabetes	3 (17)	17 (24)	0.75
Systemic Congestion	0	13 (18)	–
NYHA class III or IV	0	12 (17)	–
CAD	1 (6)	14 (20)	0.29
Severe valvular disease	0	20 (28)	<0.001
Aortic stenosis	–	4 (6)	
Aortic regurgitation	–	4 (6)	
Mitral regurgitation	–	10 (14)	
Tricuspid regurgitation	–	2 (3)	
Laboratory data			
Hemoglobin, g/dL BUN, mg/dL eGFR, mL/min/1.73 m^2^ Sodium, mEq/L logBNP, pg/mL	13 ± 1.6 15 ± 4.5 70 ± 18 141 ± 1.3 1.3 ± 0.3	12 ± 1.7 21 ± 6.4 56 ± 17 140 ± 3.0 2.3 ± 0.6	0.29 <0.001 0.004 0.01 <0.001
Medication			
ACE-I / ARB	4 (22)	42 (59)	0.007
ARNI	1（6）	14 (20)	0.29
β-blocker	3 (17)	37 (52)	0.008
Loop diuretics	0	29 (41)	–
MRA	0	28 (39)	–
SGLT2i	1 (6)	22 (31)	0.03
CCB	4 (22)	26 (37)	0.28
Echocardiography			
LVEF, %	65 ± 5.1	47 ± 17	<0.001
E/E’	9.5 ± 1.9	14 ± 5.0	<0.001
RV-FAC, %	47 ± 4.1	39 ± 8.6	0.005
TRPG, mmHg	16 ± 5.6	22 ± 11	0.03
eRAP, 3/8/15mmHg	16(89)/2(11)	55(78)/9(13)/7(10)	0.04
HV-S/D	1.7 ± 0.5	1.6 ± 1.0	0.83
Moderate/severe TR	0	12(17) / 2（3）	–
Catheterization data			
PCWP, mmHg Mean PAP, mmHg Mean RAP, mmHg	NA NA NA	12 ± 7.2 25 ± 13 4.4 ± 3.6	–
Cardiac index, L/min/m^2^	NA	2.7 ± 0.6	–

Values are means ± SD or numbers (%). *ACE-I* angiotensin-converting enzyme inhibitors, *ARB* angiotensin II receptor blocker, *ARNI* angiotensin receptor neprilysin inhibitor, *BNP* brain natriuretic peptide, *BP* blood pressure; *BUN* blood urea nitrogen, *CAD* coronary artery diseases, *CCB* calcium channel blocker, *eGFR* estimated glomerular filtration rate, *eRAP* estimated right atrial pressure, *E/E’* ratio of early diastolic peak velocity of Doppler transmitral flow to early diastolic mitral annular velocity, *HF* heart failure, *HV-S/D* ratio of hepatic vein systolic to diastolic flow velocities, *LVEF* left ventricular ejection fraction, *MRA* mineralocorticoid receptor antagonist, *NA* not available, *NYHA* New York Heart Association, *PAP* pulmonary artery pressure, *PCWP* pulmonary capillary wedge pressure, *RAP* right atrial pressure, *RV-FAC* right ventricular fractional area change ratio, *SGLT2i* sodium-glucose co-transporter-2 inhibitor, *TR* tricuspid regurgitation, *TRPG* tricuspid regurgitation pressure gradient.

### IRPI and hemodynamic parameters

**Fig. 3:**
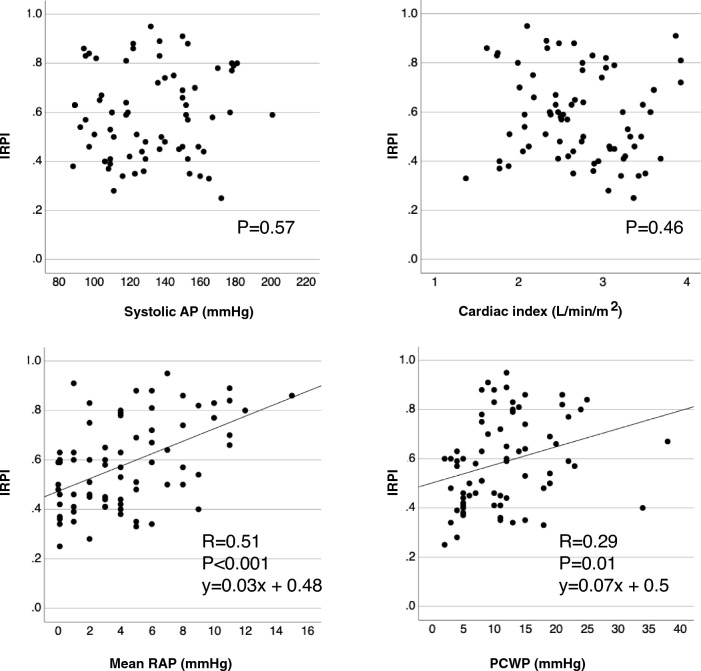
Correlations of catheterization data with IRPI in heart failure patients, *RAP*  right atrial pressure

**Fig. 4:**
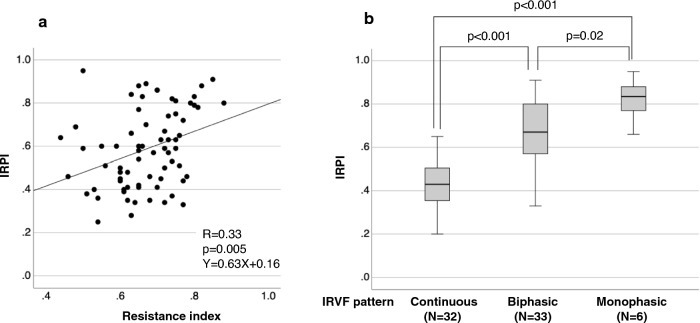
Relationship between IRPI and intrarenal Doppler profile in heart failure patients. **a**: The correlation of resistance index with IRPI. **b**: Comparison of IRPI between intrarenal Doppler flow patterns

**Fig. 5:**
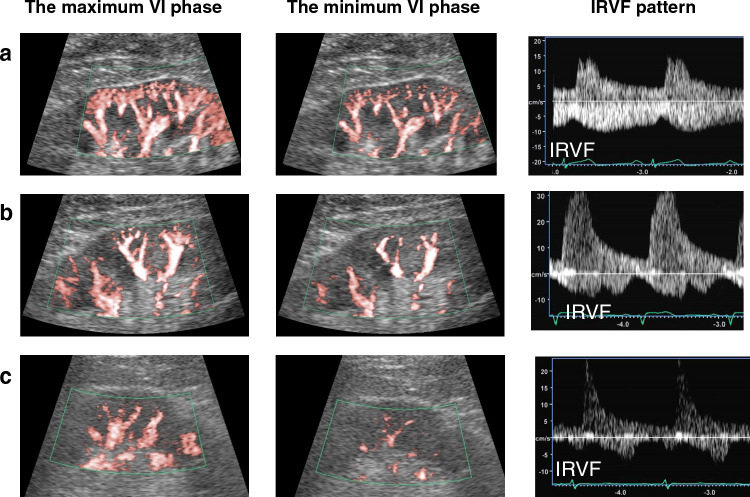
Representative images of renal superb microvascular imaging (SMI) and corresponding IRVF images. **a**: SMI in a control case. IRPI is 0.38. IRVF shows a continuous flow patten. **b**: SMI in a patient with compensated heart failure. IRPI is 0.62. IRVF shows a biphasic flow patten. **c**: SMI in a patient with decompensated heart failure. IRPI is 0.89, indicating a flash pattern. IRVF shows a monophasic flow patten

In HF patients, the correlation between catheterization data and IRPI in patients with HF is shown in Fig. 3. There was no significant correlation between IRPI and systolic AP. There was also no significant correlation between IRPI and cardiac index. In contrast, there was a significant correlation between mean right atrial pressure (RAP) and IRPI, and a significant but modest correlation between pulmonary capillary wedge pressure (PCWP) and IRPI. Figure 4 shows the association between IRPI and IRD profile. The correlation between IRPI and RI was significant but weak (*R* = 0.33, *p* = 0.005). On the other hand, IRPI varied significantly by IRVF pattern, with a higher IRPI for discontinuous IRVF patterns. Representative examples of SMI images and corresponding IRVF patterns are shown in Fig. 5.

### Characteristics of the high IRPI group

As shown in Fig. 2, IRPI was significantly higher in the HF group than in the control group; however, because of the significant overlap with the control group data, the HF group was divided into two groups, using the upper 95% CI of 0.6 for the control group as the reference value (Table 2). In patients with IRPI greater than 0.6 (IRPI > 0.6 group), the prevalence of systemic congestion, NYHA class III or IV, and biphasic or monophasic IRVF pattern was significantly higher than that in patients with IRPI less than 0.6 (IRPI ≤ 0.6 group). The IRPI > 0.6 group had higher log BNP, PCWP, mean pulmonary artery pressure, mean RAP, and RI, and lower hemoglobin and eGFR values than the IRPI ≤ 0.6 group. As shown in Table 3, multivariable logistic regression analysis identified that mean RAP and biphasic or monophasic discontinuous IRVF pattern were independent determinants of IRPI > 0.6. As seen in case c in Fig. 5, the ‘flash pattern’ of SMI is a feature in patients with higher IRPI, showing a monophasic IRVF pattern. Case b in Fig. 5 has IRPI > 0.6 and a biphasic IRVF pattern. However, whether the SMI of this example with a rather high IRPI looks like a flash pattern or not depends on subjective judgment.

**Table 2:** Comparisons between IRPI=<0.6 and IRPI>0.6 in HF group

Characteristic	IRPI=<0.6	IRPI>0.6	*P* value
	(n = 42)	(n = 29)	
Age, yrs	70 ± 10	74 ± 12	0.17
Male	31 (74)	15 (51)	0.08
Body mass index	21 ± 4.4	22 ± 4.6	0.47
Atrial fibrillation at the examination	10 (24)	9 (31)	0.60
Hypertension	24 (57)	14 (59)	1.00
Diabetes	14 (33)	3 (10)	0.046
Systemic congestion	2 (5)	11 (38)	<0.001
NYHA class III or IV	2 (5)	10 (34)	0.002
CAD	10 (24)	4 (14)	0.37
Valvular disease	19 (45)	14 (48)	0.82
Laboratory data			
Hemoglobin, g/dL	13 ± 1.7	12 ± 1.7	0.03
BUN, mg/dL	21 ± 6.5	22 ± 6.3	0.85
eGFR, mL/min/1.73 m^2^	57 ± 15	49 ± 14	0.02
Sodium, mEq/L	139 ± 3.3	140 ± 2.5	0.08
logBNP, pg/mL	2.1 ± 0.6	2.5 ± 0.5	0.005
Medication			
ACE-I / ARB / ARNI	25 (60)	17 (59)	1.00
β-blocker	24 (57)	13 (45)	0.34
Loop diuretics	17 (41)	12 (41)	1.00
MRA	19 (45)	9 (31)	0.32
SGLT2i	16 (38)	6 (21)	0.19
CCB	16 (38)	10 (35)	0.81
Echocardiography			
LVEF, %	45 ± 17	49 ± 16	0.32
E/E’	13 ± 4.7	15 ± 5.3	0.28
RV-FAC, %	40 ± 6.2	37 ± 11	0.26
TRPG, mmHg	21 ± 10	22 ± 11	0.76
eRAP, 3/8/15mmHg	35(83)/5(12)/2(5)	20(69)/4(14)/5(17)	0.03
HV-S/D	1.4 ± 0.8	2.0 ± 1.1	0.056
Moderate/severe TR	0	12(17) / 2（3）	0.86
Catheterization data			
Heart rate, beats/min	74 ± 14	75 ± 15	0.74
Systolic AP, mmHg	131 ± 27	133 ± 29	0.64
Diastolic AP, mmHg	70 ± 13	69 ± 18	0.91
Mean AP, mmHg	90 ± 15	91±18	0.75
PCWP, mmHg	9.7 ± 6.8	15 ± 7.0	0.005
Mean PA, mmHg	19 ± 3.7	27 ± 7.2	0.02
Mean RAP, mmHg	2.8 ± 2.6	6.5 ± 3.8	<0.001
Cardiac index, L/min/m^2^	2.7 ± 0.6	2.7 ± 0.6	0.88
Intrarenal Doppler			
RI	0.6 ± 0.1	0.7 ± 0.1	0.02
IRVF pattern C / B / M	30(71)/12(29)/0(0)	2(7)/21(72)/6(21)	<0.001

Values are means ± SD or numbers (%). *AP* aortic pressure; *C / B / M* continuous / biphasic / monophasic, *IRPI* intrarenal perfusion index, *IRVF* intrarenal venous flow, *RI* resistance index, other abbreviations as in Table 1

**Table 3:** Logistic regression analyses for determinants of IRPI>0.6 in HF patients

Predictor	Univariate		Multivariable	
	OR (95% CI)	*P* value	HR (95% CI)	
Systemic congestion	12.2 (2.45 – 60.9)	0.002		
NYHA class III or IV	10.5 (2.10 – 52.8)	0.004		
logBNP	4.40 (1.46 – 13.2)	0.008		
mRAP	1.42 (1.18 – 1.71)	<0.001	1.43 (1.01 – 2.04)	0.036
PCWP	1.11 (1.03 – 1.20)	0.009		
RI	4.41 (1.53 – 12.7)	0.006		
IRVF B or M pattern	32.5 (6.65 – 159)	<0.001	19.6 (3.37 – 89.9)	<0.001

B or M = biphasic or monophasic, *OR* odds ratio, other abbreviations as in Tables 1, 2.

### Determinants of eGFR

In the HF group, univariable analysis showed that mean RAP, Min.VI, and IRPI were significantly associated with eGFR (mean RAP: R = − 0.26, *p* = 0.045, Min.VI: *R* = 0.377, *p* = 0.001, IRPI: *R* = − 0.29, *p* = 0.01). Since a strong correlation was observed between IRPI and Min.VI (*R* = − 0.89, *p* < 0.001), we considered the multicollinearity between the two and analyzed them in two multivariate models: mean RAP and Min.VI, and mean RAP and IRPI. We found that Min.VI and IRPI each showed a significant association with eGFR independent of mean RAP (Min.VI: *R* = 0.37, *p* = 0.005, IRPI: R = − 0.26, *p* = 0.043). On the other hand, neither model showed a significant association with eGFR independent of mean RAP (with Min.VI: *p* = 0.46, with IRPI: *p* = 0.43).

Intra- and inter-observer variability of Max.VI, Min.VI, and IRPI measurements were as follows; Max.VI: 8.0 ± 4.6 and 8.5 ± 8.0 (%), Min.VI: 18.4 ± 14.0 and 14.7 ± 13.9 (%), IRPI: 10.4 ± 6.1 and 8.9 ± 10.3 (%), respectively.

## Discussion

This prospective single-center study showed that IRPI, a parameter related to SMI signal variability during the cardiac cycle, may represent intrarenal hemodynamics associated with congestive HF. In this cohort, IRPI was identified as an independent determinant of eGFR along with Min.VI. The simultaneous catheterization and ultrasound studies were an advantage of this study and support the reliability of the findings.

SMI allowed us to visualize the circulation in the kidney. While IRVF waveforms and RI from IRD are methods to evaluate renal circulation from local blood flow in the kidney, SMI is unique in that it can visualize blood flow within a wide ROI within the kidney. The measure of VI used in SMI quantifies the blood flow rate moving within an ROI at a given time phase. Decreased intrarenal blood flow due to impaired intrarenal circulation is thought to reduce VI, and this study found that both Max.VI and Min.VI were lower in the HF group than in the control group. In addition, since previous studies have shown that the IRD signal fluctuates in the cardiac cycle and that this fluctuation is more significant in renal circulatory failure, it was expected that IRPI, the index of variation of the VI, would be more significant in intrarenal circulatory failure. In fact, in this study, there were more systemic congestive signs and more elevated BNP levels, and catheter data suggestive of advanced HF in the group with higher IRPI (> 0.6), which could prove that assumption.

Renal blood flow is assumed to be involved not only in congestion of the venous system but also in inflow from the arteries. This is because, in clinical practice, low cardiac output is thought to strongly influence renal perfusion in HF patients. We hypothesized that intrarenal blood flow dynamics may need to consider arterial hemodynamics. Taking advantage of the fact that SMI cannot detect the directionality of blood flow and distinguish between arterial and venous blood flow for quantification, we thought that IRPI could provide information on both arterial and venous blood flow, allowing us to test our hypothesis and verify its validity. However, contrary to expectations, arterial hemodynamic variables, including cardiac output, blood pressure, and RI, had little effect on IRPI compared to venous hemodynamic variables, including mean RAP and IRVF pattern. The results support the notion that the presence of a low-flow state due to HF only partially explains the pathophysiology of CRS. According to the ADHERE registry, the incidence of elevated serum creatinine is similar in patients with reduced or preserved systolic function in acute decompensated HF [16]. Furthermore, most patients admitted with acute CRS have normal blood pressure and normal LVEF [17]. It is important to maintain a sufficient pressure differential between arterial driving pressure and venous outflow pressure for adequate renal blood flow and glomerular filtration [18]. Damman et al. [19] demonstrated that renal blood flow (RBF) measured using constant infusion of radiolabeled tracers is the main factor determining GFR in patients with cardiac dysfunction and increased RAP. Our study suggests that IRPI or Min.VI may also be an indicator that can be used to estimate RBF, but the significant correlation between IRPI or Min.VI and eGFR in this study is weaker than the correlation between RBF and GFR demonstrated by Daman et al. [19]. This difference in the strength of the correlation with GFR is mainly due to the characteristics of each indicator. However, it may also be influenced by differences in the HF status of the patients studied and the method used to measure GFR.

As previous studies have reported that IRVF patterns, a known indicator of renal congestion, suggest more advanced HF pathology and are associated with poorer clinical prognosis than RI, an indicator of the arterial system, this study reveals that IRPI is also more strongly dependent on blood flow in the renal venous system. These findings suggest that IRVF pattern is relatively representative of the renal circulation, even though it is a method of assessing regional renal blood flow. However, the wide distribution of IRPI in the biphasic IRVF pattern group is an important finding in this study. In other words, IRPI may be able to detect high-risk cases in the biphasic IRVF pattern group, suggesting the superiority of SMI over IRD in assessing renal blood flow.

Because IRVF is an imaging evaluation method that can be performed at a local renal site, i.e., the renal interlobar vein, it may not be possible to obtain images worthy of evaluation depending on anatomical limitations and the examination environment. For example, very severe renal congestion and reduced renal perfusion may result in highly reduced venous velocity and failure to capture the pulsed Doppler signal. However, SMI is excellent at detecting blood flow at low velocities and may be able to assess renal perfusion in such situations. Also, if the patient is unwell and has difficulty holding their breath during the examination, the pulsed Doppler technique cannot be applied to capture regional blood flow in the renal interlobar veins because of respiratory displacement of the kidneys, whereas SMI, which uses blood flow signals throughout the kidney, has fewer such limitations. In this regard, SMI has the potential to image the renal circulation more easily and may be a valuable alternative to IRVF. In cases of renal congestion, SMI varies so dramatically that it resembles a flash of light, as shown in case c in Fig. 5. The ability to assess renal circulation based on these characteristic findings, without relying on the quantitative measure IRPI, would be a major advance in the clinical application of SMI. To be able to judge these phenomena subjectively, one would need to experience a variety of cases, but it would not be difficult and would not require any special skills. On the other hand, in cases with mildly elevated IRPI, as in case b in Fig. 5, the extent to which the image is flash-like depends on subjective perception. Therefore, quantification using IRPI is unavoidable in cases other than the typical cases such as case c in Fig. 5. In addition, sensing changes in SMI by observing specific cases over time would be helpful in gaining sensory SMI assessment skills. However, even in such cases, it may be necessary to combine this with quantification methods to determine if the image is no longer a flash-like image and if the IRPI is less than 0.6. Overcoming these challenges is an important issue for the use of SMI in clinical practice, and future research is needed.

### Limitations

This study was performed at a single center, and the study population was small and comprised HF patients whose condition was relatively stable; therefore, further investigations are needed to determine the feasibility and reliability of SMI in clinical practice.

In addition, the clinical value of applying SMI to the treatment of HF needs to be scrutinized. Since this study was cross-sectional, the use of IRPI values, especially the treatment of renal and systemic congestion aiming at IRPI values less than 0.6, should be clarified to determine what impact it has on renal function and clinical outcome in patients with HF in a large-scale multicenter study.

This study also showed that hemodynamic parameters alone could not fully explain impaired renal circulation. Therefore, further investigations are needed to explore other mechanisms induced by impaired renal circulation, such as activations in the renin–angiotensin–aldosterone system and sympathetic nervous system, chronic inflammation, vascular endothelial dysfunction, renal interstitial fibrosis, and anemia [1].

## Conclusion

Flash-like variations in the SMI signal during the cardiac cycle are characteristic of impaired renal circulation in HF. Its quantitative parameter IRPI can be an evaluation method for renal perfusion impairment in HF. IRPI may also be an alternative evaluation method for renal congestion primarily because of its strong dependence on IRVF patterns and independent association with RAP.
